# RNA-Seq reveals MicroRNA expression signature and genetic polymorphism associated with growth and muscle quality traits in rainbow trout

**DOI:** 10.1038/s41598-017-09515-4

**Published:** 2017-08-22

**Authors:** Bam Dev Paneru, Rafet Al-Tobasei, Brett Kenney, Timothy D. Leeds, Mohamed Salem

**Affiliations:** 1 Department of Biology and Molecular Biosciences Program, Middle Tennessee State University, Murfreesboro, TN 37132 United States; 2 Computational Science Program, Middle Tennessee State University, Murfreesboro, TN 37132 United States; 3 Division of Animal and Nutritional Science, West Virginia University, Morgantown, 26506-6108 West Virginia United States; 4 The National Center for Cool and Cold Water Aquaculture, USDA Agricultural Research Service, Kearneysville, WV 25430 United States

## Abstract

The role of microRNA expression and genetic variation in microRNA-binding sites of target genes on growth and muscle quality traits is poorly characterized. We used RNA-Seq approach to investigate their importance on 5 growth and muscle quality traits: whole body weight (WBW), muscle yield, muscle crude-fat content, muscle shear force and whiteness. Phenotypic data were collected from 471 fish, representing 98 families (~5 fish/family) from a growth-selected line. Muscle microRNAs and mRNAs were sequenced from 22 families showing divergent phenotypes. Ninety microRNAs showed differential expression between families with divergent phenotypes, and their expression was strongly associated with variation in phenotypes. A total of 204 single nucleotide polymorphisms (SNPs) present in 3′ UTR of target genes either destroyed or created novel illegitimate microRNA target sites; of them, 78 SNPs explained significant variation in the aforementioned 5 muscle traits. Majority of the phenotype-associated SNPs were present in microRNA-binding sites of genes involved in energy metabolism and muscle structure. These findings suggest that variation in microRNA expression and/or sequence variation in microRNA binding sites in target genes play an important role in mediating differences in fish growth and muscle quality phenotypes.

## Introduction

MicroRNAs are important post-transcriptional regulators of genes. In humans, about one-third of the genes are regulated by microRNAs^1^, which suggests an important regulatory role of microRNAs in gene expression and hence phenotype determination^2^. There is evidence that a single microRNA can regulate hundreds of genes whereas the same gene can be regulated by hundreds of microRNAs^3^. The seed region in mature microRNA sequence, usually extending from 2–7 nts at the 5′ end, binds to the microRNA recognition element seed site (MRESS) in 3′ UTR of target gene, and plays a vital role in determining specificity of microRNA-mRNA binding^1^. This ‘microRNA-target mRNA’ binding leads to downregulation of the gene by various mechanisms such as translation suppression^4^, target mRNA cleavage^5^ and deadenylation^6^. Therefore, any mutation that either destroys or creates a novel illegitimate MRESS in target genes have important functional consequences in phenotype^7, 8^.

MicroRNA mediated gene regulation plays critical role in embryonic myogenesis as well as post-embryonic skeletal muscle growth. Myogenic microRNAs mir-1, mir-133 and mir-206 control skeletal muscle growth by directly or indirectly regulating genes involved in myogenesis(see review^9^). Loss of mir-206 function in Nile tilapia leads to accelerated muscle growth because insulin-like growth factor-1 is a direct target of mir-206^10^. In zebrafish, mir-1 and mir-133 are responsible for more than half of the microRNA-mediated gene regulation in muscle^11^. Non-muscle specific microRNAs also regulate different aspects of myogenesis and muscle development in fish. As an example, mir-143 and mir-203b target myoD, a member of myogenic regulatory factors (MRFs), in Mandarin fish and Nile tilapia, respectively^12, 13^. A novel zebrafish microRNA, mir-In300, targets dickkopf-3 (*dkk3*) gene and hence abolishes the promoter activity of myogenic protein 5 (myf5)^14^. Similarly, zebrafish mir-214 controls hedgehog signaling mediated speciation of muscle cell by regulating suppressor of fused *(sufu)* gene^15^. Further, Let-7, mir-19 and mir-130 show regulated expression during transition from muscular hyperplasia to hypertrophy in fish^16^. Despite these aforementioned studies, there is still need for complete microRNAome expression and genetic variant profiles to understand the genetic basis of variation in muscle growth and quality traits in food fish production.

Scientists at the USDA, ARS National Center for Cool and Cold Water Aquaculture (NCCCWA) have developed a pedigreed line of rainbow trout selectively bred for 5 generations for improved growth performance to the standard US market body weight and beyond^17^ and have characterized muscle yield and quality traits in nucleus families within the line. The objectives of this study were to 1) investigate association of microRNA expression with muscle growth and muscle quality traits in the NCCCWA growth-selected line and 2) investigate effects of single nucleotide polymorphisms (SNPs) in microRNA binding sites on growth and muscle quality traits. Using high throughput deep small RNA sequencing approach, we identified differentially expressed (DE) microRNAs between fish families showing contrasting phenotype for whole-body weight (WBW), muscle yield, crude fat content, shear-force and whiteness of the muscle. We performed ‘phenotype-microRNA expression’ association using a random sample of 90 fish from 3^rd^-generation families (2010 hatch year) to investigate potential impact of microRNA expression to the phenotype in the population. SNPs capable of creating or destroying microRNA binding sites in protein coding target genes were identified and their functional consequence on growth and muscle quality phenotypes was evaluated by ‘SNP-phenotype’ association analysis using a sample of 786 fish from 3^rd^- and 4^th^-generation families (2010 and 2012 hatch years).

## Result and Discussion

### Muscle trait phenotypes and Small RNA sequencing

**Figure 1:**
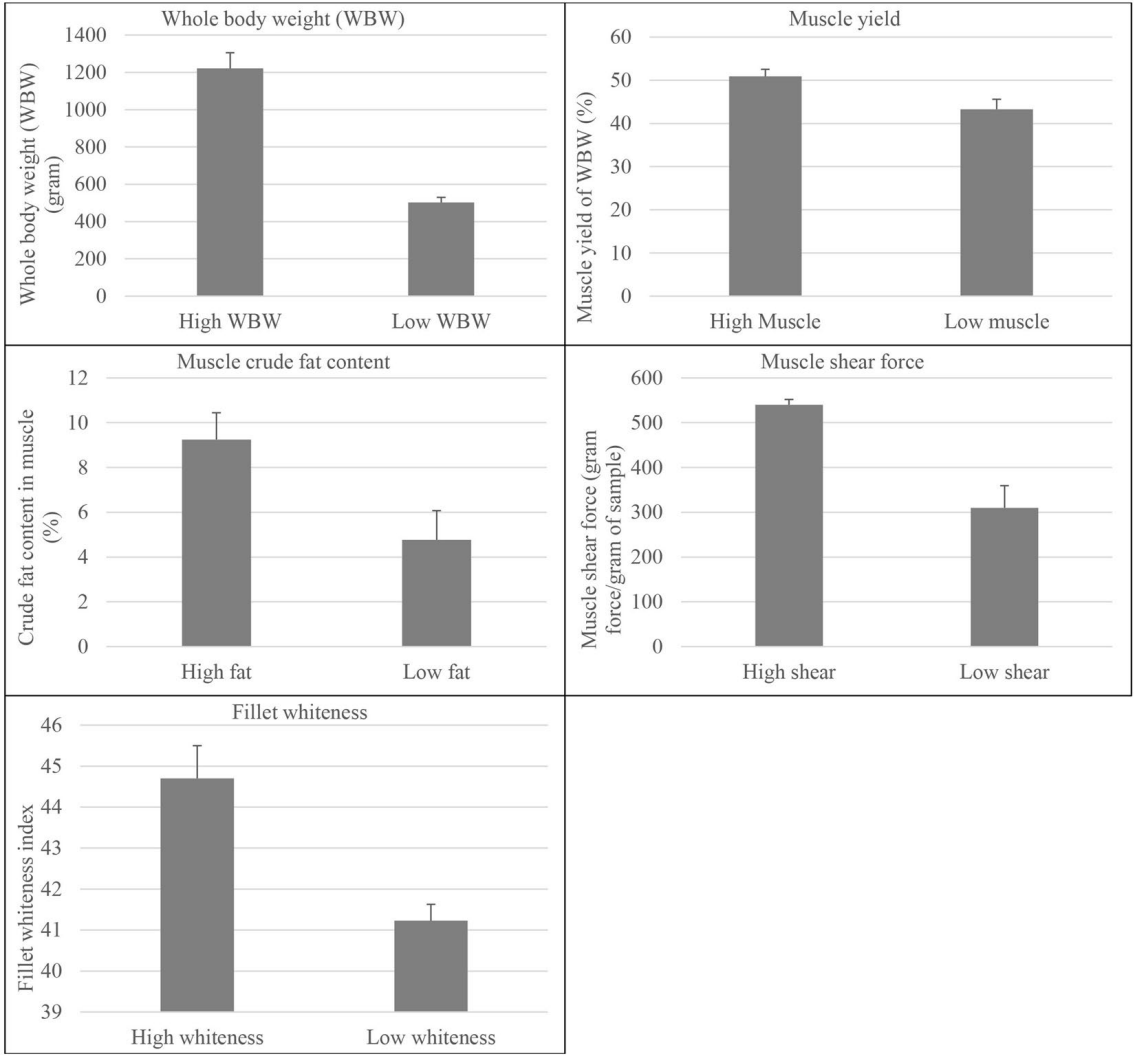
Phenotype comparison of high and low ranked families of growth and muscle quality traits. Phenotypic difference for whole body weight (WBW) and 4 muscle quality traits (muscle yield, crude fat content, shear force and whiteness index) of top 4 high ranked and 4 low ranked families (5 fish/family) of selectively-bred trout at ~13-month post-hatch. Phenotypes were statistically different between high ranked and low ranked families (P < 0.01). Error bars on graph show standard deviation.

Phenotypic divergence between the 4 highest-ranked and 4 lowest-ranked families for the 5 traits were: WBW (1221.6 g ± 84.25 vs. 502.1 ± 28.0 g); muscle yield (50.9% ± 1.6 vs. 43.3% ± 2.3); crude-fat (9.24% ± 1.2 vs. 4.77% ± 1.3); shear force (grams force/grams of sample; 539.64 ± 12.3 vs. 310.01 ± 49.2); and muscle/fillet whiteness index (44.7 ± 0.8 vs. 41.23 ± 0.4) (Fig. 1). Since some fish families were common between the traits, the total number of sequenced families was 22 (Table 1).

**Table 1:** Small RNA sequencing and annotation statistics of 22 samples used in the study.

Fish family	Read count and annotation statistics		Putative microRNA count and annotation statistics		
	Number of raw reads	Number of reads after trimming and filtration	Total number putative microRNAs detected	Putative microRNAs annotated to Mirbase: Count (Percentage)	Number of non-redundant annotated microRNAs
195^i^	11,168,118	9,881,880	150,902	35,355 (23.4%)	1,221
262^ehi^	14,841,753	12,357,559	318,231	42,577 (13.4%)	1,335
277^e^	11,012,983	8,522,071	160,763	31,794 (19.8%)	1,188
366^fb^	10,352,049	7,551,995	149,061	31,321 (21.0%)	1,212
390^eia^	13,682,126	12,138,640	219,575	42,283 (19.3%)	1,277
399^hb^	10,313,768	7,804,634	168,925	31,952 (18.9%)	1,194
405^i^	10,309,353	7,979,759	145,673	31,256 (21.5%)	1,173
556^dfhb^	9,954,340	8,203,301	146,394	32,682 (22.3%)	1,205
565^d^	10,384,499	7,709,062	189,110	30,388 (16.1%)	1,236
580^e^	7,490,428	4,261,515	147,037	24,980 (17.0%)	1,115
595 ^g^	12,040,061	8,372,165	319,839	35,112 (11.0%)	1,209
191^cj^	21,246,629	18,766,660	247,811	30,175 (12.2%)	1,092
193^gja^	25,361,890	19,439,379	141,126	35,160 (24.9%)	1,121
201^cja^	12,676,918	11,296,840	138,647	21,561 (15.6%)	989
357^c^	19,472,380	14,507,911	251,792	29,545 (11.7%)	1,054
408 ^g^	13,845,919	12,152,718	94,556	21,053 (22.3%)	948
51^hi^	20,964,015	17,185,620	241,354	37,891 (15.7%)	1,246
559^dfjb^	13,714,117	11,099,365	136,184	18,551 (13.6%)	966
593^d^	23,474,784	19,593,896	573,692	42,111 (7.3%)	1,261
597^c^	22,048,522	20,079,565	255,680	33,440 (13.1%)	1,160
65^e^	15,949,419	13,873,696	103,005	22,097 (21.5%)	998
194^a^	9,115,039	7,974,575	138,450	31,695 (22.9%)	1,189

Table shows name of fish families used for different traits, number of raw reads, number of reads after trimming/filtration, total number of putative microRNAs detected in each sample and number of putative microRNAs annotated to the miRBase microRNA reference. Last column represents the number of non-redundant microRNAs annotated to MiRBase microRNA reference in each sample. Note: ^a^High WBW, ^b^Low WBW, ^c^High muscle yield, ^d^Low muscle yield, ^e^High crude fat, ^f^Low crude fat, ^g^High shear, ^h^Low shear, ^i^High muscle whiteness, ^j^Low muscle whiteness and families used for more than one muscle traits are indicated with corresponding multiple superscripts.

High throughput small RNA sequencing resulted into mean sequencing depth of 14.5 million reads per sample. After trimming of sequencing adaptor, average length of reads was 22 nucleotides (Supplementary Dataset 1A), which is a typical length average for mature microRNAs. After filtration and adapter trimming, the average number of reads in each sample was 11.9 million. On average, ~0.2 million potential microRNA transcripts were detected from trimmed reads in each sample. Of these potential microRNA transcripts, ~17.5% had sequence homology with mature microRNAs in the miRBase database. From these annotated microRNAs in each sample, different variants of the same microRNA were collectively counted as a single microRNA, which resulted into an average of 1,154 unique microRNA per sample (Table 1).

RNA-Seq based principal component analysis (PCA) of 22 families sequenced for gene expression study showed no cluster suggesting genetic divergence within the population (Supplementary Dataset 1B).

### Differentially expressed microRNAs between high and low ranked families for growth and muscle traits

**Figure 2:**
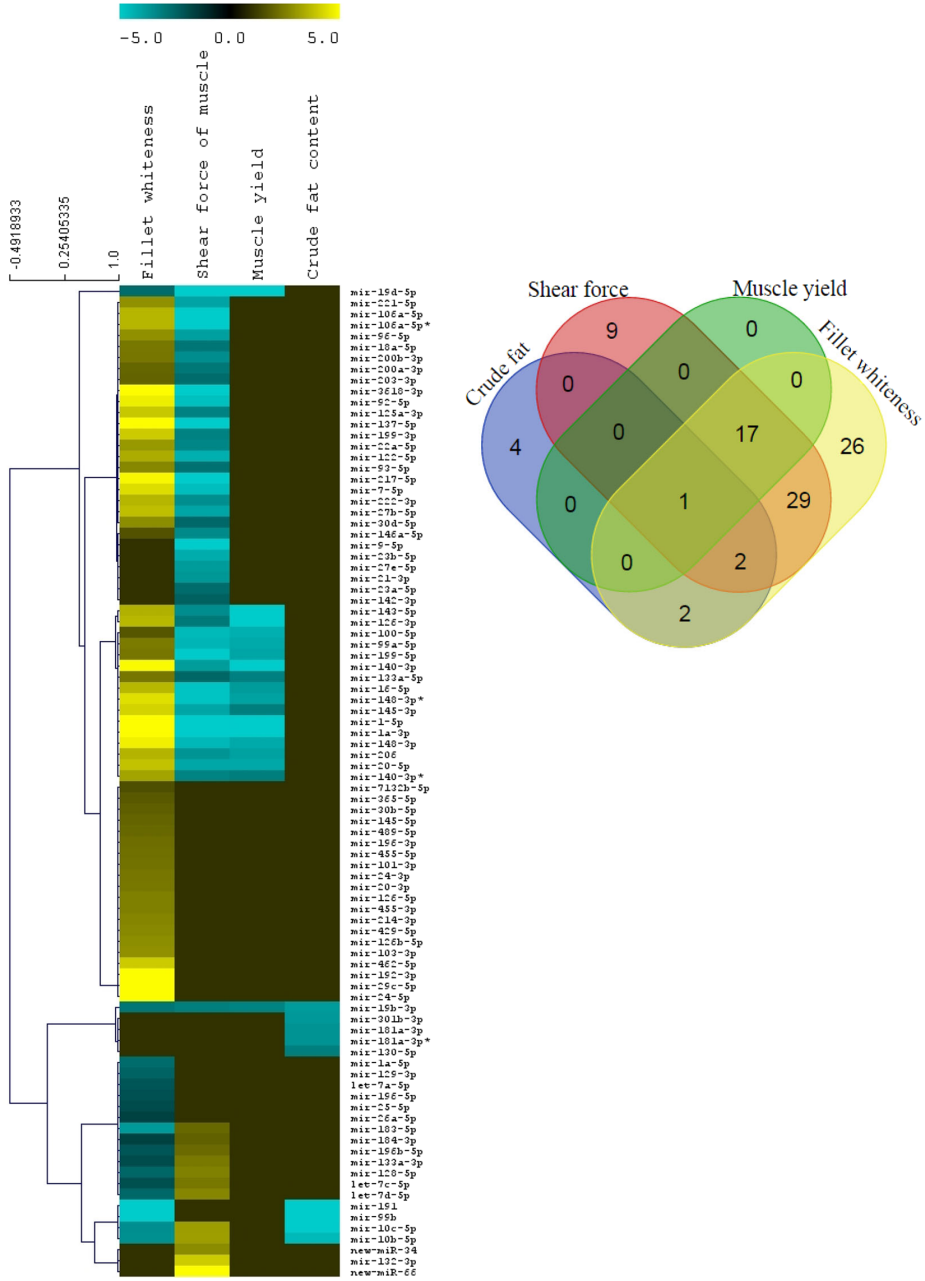
Heat map of fold change of differentially expressed (DE) microRNAs between high vs low ranked families of various traits (left) and Venn-diagram showing shared DE microRNAs between different traits (right). In heat map, dark green and yellow colors indicate downregulation and upregulation respectively in high ranked family compared to low ranked family. Dark color indicates no differential expression of microRNAs. Note that the value of color limit (−5 to 5) does not reflect true fold change as values of fold change were transformed (log2) and color scale was adjusted to make the heat map more visible. True fold change is given in Supplementary Dataset 1B.

In order to identify microRNAs associated with growth and muscle quality traits, we profiled DE microRNAs between families with divergent phenotypes. None of the microRNAs were DE in association with WBW. However, 90 microRNAs were DE in remaining four muscle traits (Fig. 2 and Supplementary Dataset 1C). Two of the DE microRNAs were recently discovered microRNAs in rainbow trout; new-miR-66 and new-miR-34^18^. A total of 18, 9, 56 and 77 microRNAs were DE between high and low ranked families for muscle yield, crude fat content, shear force and muscle whiteness, respectively. Most of the DE microRNAs were shared between several phenotypic traits (Fig. 2 [Venn diagram] and Supplementary Dataset 1C). For example, mir-19b was DE in all four muscle traits. All 18 DE microRNAs in muscle yield families were also DE in shear force and muscle whiteness families. Similarly, out of 56 DE microRNAs in shear force families, 49 were also DE in association with muscle whiteness. This observation suggests that common mechanisms including coordinated expression of microRNAs may affect muscle yield and quality traits in rainbow trout. This argument is supported by previous report pinpointing to interrelated nature of these muscle traits in fish ^19^.

Interestingly, the direction of change of the shared DE microRNAs were consistently either positively or negatively correlated between traits. For example, all 18 DE microRNA that were downregulated in association with increased muscle yield were also downregulated in families showing high shear force (R^2^ = 0.54) (Fig. 2 [heat map] and Supplementary Dataset 1C). In contrast, most of these downregulated microRNAs in families with high muscle yield (16 out 18) were upregulated in the high-whiteness families (R^2^ = −0.56). In addition, 47 out of 49 of the shared DE microRNAs between the shear force and muscle whiteness groups showed opposite pattern of differential expression (R^2^ = −0.87). Similarly, all 5 shared DE microRNAs between crude fat and whiteness groups were downregulated in high ranked families of both traits (R^2^ = 0.87). Out of 3 DE shared microRNAs between shear and fat group, 2 microRNAs showed opposite fold change pattern between the traits. In accordance with this observation, growth and muscle quality phenotypes of 500 fish population used in this study showed correlation between traits (Table 2). WBW showed positive correlation with muscle yield (R^2^ = 0.32, p < 0.0001) and crude fat content (R^2^ = 0.33, p < 0.0001). Similarly, muscle yield showed very weak but significant positive correlation with crude fat (R^2^ = 0.061, p < 0.0001) and shear force (R^2^ = 0.03, p = 0.0003), and negative correlation with whiteness (R^2^ = −0.023, p = 0.0009). Crude fat content and whiteness had weak but positive correlation (Table 2). This finding suggests that correlation among phenotypic traits could be, at least partially, explained by variation in expression level of DE microRNAs. Consistent with this observation, a recent report in salmon has indicated that crude fat content is negatively correlated with muscle shear force and positively correlated with L* (lightness) and b* (yellowness) of raw salmon muscle^19^.

**Table 2:** Pairwise comparison showing correlation between different growth and muscle quality phenotypes.

	Whole Body Weight (WBW)	Muscle yield	Muscle shear force	Muscle whiteness
Muscle yield	0.323 (<0.0001)			
Muscle shear force	0.045 (<0.0001)	0.030 (0.0003)		
Muscle whiteness	0.001 (0.530)	−0.023 (<0.0001)	−0.030 (0.0002)	
Muscle crude fat content	0.328 (<0.0001)	0.061 (<0.0001)	0.00001 (0.946)	0.050<0.0001)

Correlation was calculated from phenotypic data of ~500 individual fish of USDA-ARS-NCCCWA’s growth selected line used in this study. Table shows correlation coefficients (R^2^) between each pair of traits and p value inside the brackets. Negative (−) value indicates negative correlation.

### Targets of DE microRNAs and their functional annotation

**Figure 3:**
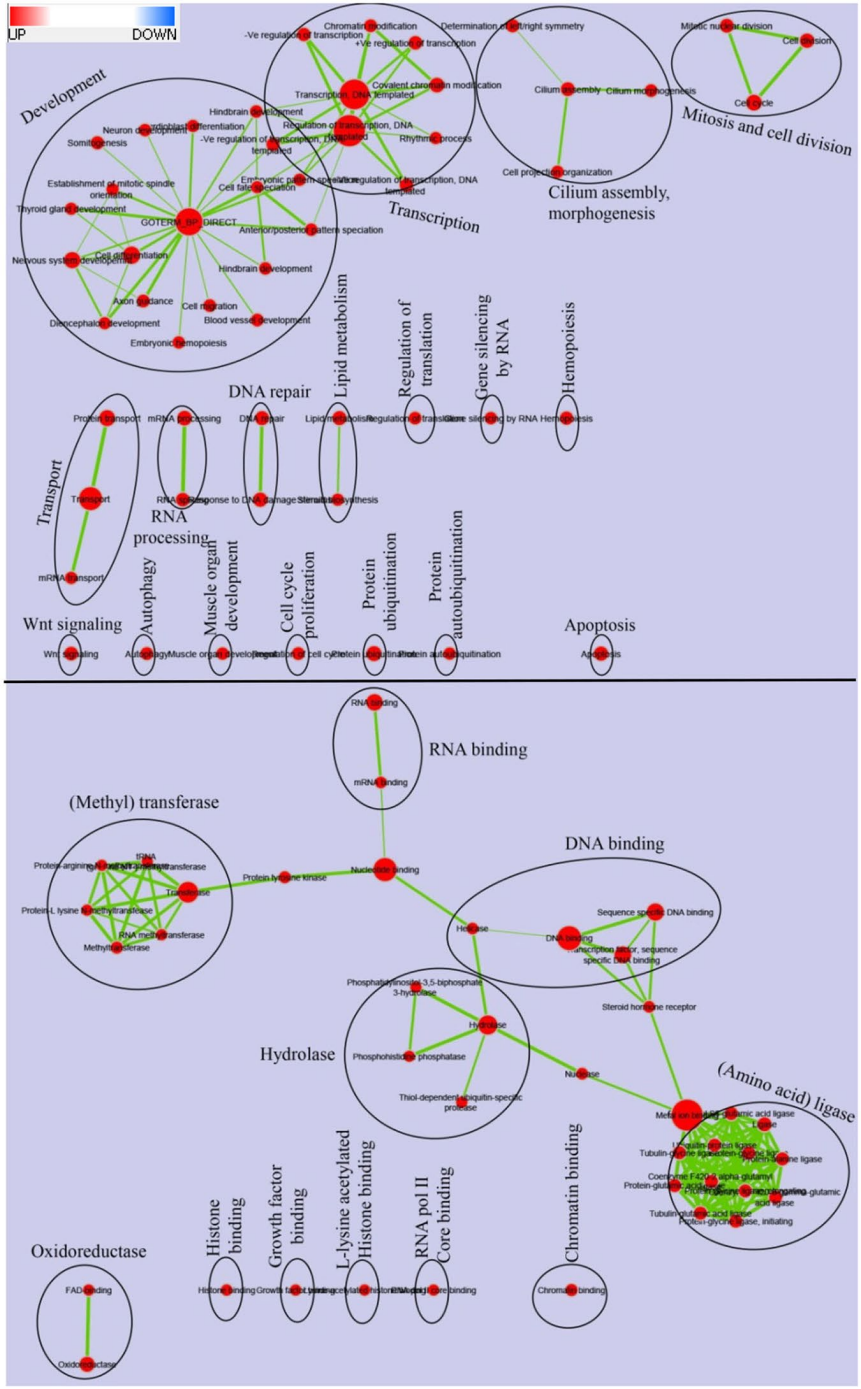
Enrichment map and enriched gene pathways of predicted microRNA targets classified into the biological process (top) and molecular function (bottom) categories. In enrichment map, enriched gene-sets represent nodes, which are related/connected by their GO relation(s) (edges). Red node color represents enriched gene-set. Color intensity of the node represents significance of enrichment; node size represents number of genes in the gene-set (proportional relation) and edge thickness represents degree of overlap between gene-sets (proportional relation). Note: Same node size in molecular function and biological process category does not include the same number of genes because maps are generated separately and then combined.

A total of 6,837 protein-coding genes were identified as potential targets with high confidence for 90 DE microRNAs (Supplementary Dataset 2). To investigate the functional significance of the predicted target genes, we performed gene enrichment analysis (GEA) using DAVID^20, 21^. A list of all overrepresented pathways in different GO categories are provided in Supplementary Dataset 3. As shown in Fig. 3, in the biological process category, genes involved in multicellular organism development (4.2-fold), transcription (3.7-fold) and mitosis/cell division (4.0-fold) were highly overrepresented. Other significantly enriched pathways included RNA processing, DNA repair, gene silencing by RNA, protein ubiquitination, lipid metabolism, muscle organ development, regulation of growth, cell proliferation and apoptosis. Similarly, among the signal transduction pathways, Wnt signaling pathway was overrepresented, which is involved in skeletal muscle growth/myogenesis^22–24^ and regulation of growth control genes^25^.

In the molecular function pathways category, most of the overrepresented gene sets had functions related to epigenetic gene regulation such as methyltransferase activity, histone binding and chromatin binding activity (Fig. 3). Methyltransferases are important in epigenetic gene-regulation and are known to regulate skeletal muscle growth by regulating expression of myoD transcription factor^26^. Another enriched gene set included various ligases that post translationally modify proteins by adding specific amino acid; e.g. protein-glycine ligases, protein-glutamic acid ligase and tubulin-glycine ligase. These findings suggest that DE microRNAs may control growth and muscle quality phenotypes primarily via post-transcriptional regulation of genes involved in development, muscle growth as well as the genes involved in epigenetic gene regulation and protein modification.

### MicroRNAs associated with growth and muscle quality traits

To estimate the association between microRNA expression and variation in phenotypes, 12 highly and commonly (among traits) DE microRNAs were selected for ‘microRNA expression-phenotype’ regression analysis. Expression level of microRNA was qPCR-quantified and correlated to phenotypes in 90 randomly selected fish from the same population. Out of the 12 microRNAs, 10, 12, 10, 5 and 6 correlated with WBW, muscle yield, crude fat content, shear force and muscle whiteness respectively (cut off: R^2^ > 0.05, p-value < 0.05) (Table 3). When the effect of all 12 microRNA expression levels was combined, about 31%, 42%, 22%, 13% and 26% variation in WBW, muscle yield, muscle crude fat, muscle shear force and muscle whiteness, respectively, was explained by variation in microRNA expression.

**Table 3:** Correlation between microRNA expression level and phenotypic variation for different muscle quality traits.

DE microRNAs	Correlation with WBW (p value)	Correlation with muscle yield (p value)	Correlation with muscle crude fat content (p value)	Correlation with muscle shear force (p value)	Correlation with muscle whiteness (p value)
mir-1a-3p	−0.165 (0.002)	−0.188 (0.007)	−0.075 (0.031)	NA	NA
mir-126-3p	NA	−0.068 (0.032)	NA	−0.065 (0.049)	0.076 (0.003)
mir-19b-3p	−0.072 (0.03)	−0.172 (0.0005)	−0.090 (0.013)	NA	NA
mir-148-3p*	−0.134 (0.002)	−0.150 (0.001)	−0.070 (0.030)	−0.090 (0.024)	0.087 (0.019)
mir-20-5p	−0.103 (0.008)	−0.183 (0.0003)	−0.081 (0.019)	−0.073 (0.033)	0.088 (0.018)
mir-206	−0.084 (0.02)	−0.140 (0.002)	NA	−0.072 (0.04)	0.084 (0.022)
mir-133a-5p	−0.110 (0.006)	−0.158 (0.001)	−0.138 (0.001)	−0.070 (0.04)	0.065 (0.042)
mir-143-5p	−0.0730 (0.030)	−0.130 (0.001)	−0.073 (0.028)	NA	0.061 (0.049)
mir-99b	−0.056 (0.052)	−0.110 (0.010)	−0.122 (0.004)	NA	NA
mir-10c-5p	−0.100 (0.010)	−0.082 (0.018)	−0.063 (0.040)	NA	NA
mir-10b-5p	NA	−0.067 (0.034)	−0.081 (0.021)	NA	NA
mir-181a-3p	−0.078 (0.022)	−0.100 (0.009)	−0.066 (0.035)	NA	NA

Correlation was performed based on real time PCR quantification of microRNAs in 90 random individual fish and their phenotypic measurements for different traits. Negative value (−) indicates negative correlation.

MicroRNAs associated with each phenotypes and their relevant protein coding target genes are described in separate section below.

#### Whole body weight (WBW)

No microRNA was DE between the high and low ranked families of WBW phenotype. However, microRNA expression-phenotype regression analysis performed on 90 randomly sampled fish showed that variation in expression of 10 DE microRNAs explained significant variation in WBW (Table 3). These microRNAs included muscle specific myogenic microRNAs as well as other microRNAs that were DE in response to muscle yield and/or muscle crude fat content. Expression of individual microRNA explained 5.6% to 16.5% variation in WBW depending on the microRNA (Table 3). We calculated the fold change between high vs low ranked WBW families for these 10 microRNAs based on qPCR data. Majority of these microRNAs showed small but significant fold change between high vs low ranked WBW families. It is possible that RNA-Seq failed to detect such a small change in fold change, which qPCR did. Future mechanistic study involving gene knockout or dose-dependent response of individual microRNA on WBW may help understand biology of WBW growth.

#### Muscle yield

There were a total of 18 microRNAs downregulated in the high muscle yield families compared to their low muscle yield counterparts (Fig. 2 and Supplementary Dataset 1C). Of them, expression pattern of 12 microRNAs including mir-1, mir-133 and mir-206 was validated by real time PCR, which showed consistency between qPCR and RNA-Seq approaches (Supplementary Dataset 1D). In addition, all 9 DE microRNAs that were chosen for phenotype-genotype regression analysis, negatively correlated with muscle yield (p < 0.05). Variation in expression level of each microRNA explained 6.8% to 18.8% of the variation in muscle yield, with the most significant microRNA being mir-1a-3p (Table 3). Although expression of mir-10c-5p and mir-181a-3p was not statistically different in the RNA-Seq data between high and low ranked muscle yield groups, their expression was significantly and negatively correlated with muscle yield based on qPCR quantification in random 90 individuals (Table 3). To the best of our knowledge, 8 out of 18 DE microRNAs (mir-19d, mir-100–5p, mir-99a-5p, mir-148-3p, mir-199-5p, mir-148-3p*, mir-19b-3p and mir-140) were not reported before as associated with muscle growth. The remaining 10 DE microRNAs are known to directly or indirectly regulate skeletal muscle development in different species by regulating genes involved in myogenesis^12, 27^, muscle-growth-related signal transduction pathways^9, 28, 29^ and cell cycle^30^. As an example, mir-1 and mir-133 are responsible for more than half of the microRNA-mediated gene regulation in muscle of zebrafish^11^. Similarly, mir-143 regulates expression of myoD in skeletal muscle of fish^12^. These findings suggest that differential gene expression has identified several microRNAs previously known to regulate myogenesis as well as several additional microRNAs potentially implicated in muscle growth.

A total of 1,743 protein-coding genes were predicted as potential targets of 18 downregulated microRNAs in high muscle yield group (Supplementary Dataset 2). Interestingly, 10 significantly enriched gene pathways in the target gene list were directly involved in muscle growth e. g. muscle organ development (4.4-fold), skeletal muscle tissue development (4.5-fold) and muscle cell differentiation (3.1-fold) (FDR-p < 0.05) (Supplementary Dataset 4). Other enriched gene pathways included chromatin modification (5.4-fold), transcription (3.2-fold), cell cycle (4.7-fold), and multicellular organism development (4.6fold). These findings suggest that DE microRNAs may regulate muscle yield by regulating genes directly involved in skeletal muscle development, transcription, cell cycle, and/or protein degradation^12, 27, 29, 30^.

#### Crude fat content in muscle

A total of 9 microRNAs were significantly downregulated in the high-fat fish families compared to low-fat families (Fig. 2 and Supplementary Dataset 1C). Seven out of 9 DE microRNAs are well documented as associated with adipogenesis and/or body fat deposition in different species^31–35^. The remaining 2 DE microRNAs (mir-10c-5p and mir-19b-3p) were not reported before, to the best our knowledge. A total of 494 protein-coding genes were predicted as potential target of the 9 DE microRNAs (Supplementary Dataset 2). Four biological pathways multicellular organism development (4.7-fold), cell differentiation (4.1-fold), transcription (3.3-fold) and regulation of transcription (3.1-fold) were significantly enriched in the target gene list (FDR-p < 0.05) (Supplementary Dataset 4). Mir-10c-5p and mir-19b-3p, whose association with adipogenicity was previously unknown, targeted several genes involved in adipocyte differentiation (e. g. suppressor of cytokine signaling 6), fat storage (e. g. perilipin-2 isoform), lipid metabolism (e. g. peroxisomal fatty acyl-coA oxidase-1, lysophosphatidylcholine acyltransferase 2) and lipid transport (e. g. apolipoprotein b-100 and microsomal triglyceride transfer protein) (Supplementary Dataset 2).

Three of the nine microRNAs (mir-10c-5p, mir-10b-5p and mir-181a-3p) were qPCR analyzed for association with variation in muscle fat content in 90 individual fish. The regression analysis showed that these microRNAs explained 6.3% to 8.1% of the variation in fat content in muscle (Table 3). In addition to these 3 DE microRNAs, 7 non-DE microRNAs that were associated with muscle yield (mir-1a-3p, mir-19b-3p, mir-148-3p*, mir-20-5p, mir-133a-5p, mir-143-5p and mir-99b) were also significantly correlated with muscle crude fat content (FDR-p < 0.05), (Table 3). These findings suggest that DE microRNAs may play crucial role in post-transcriptional regulation of genes associated with crude fat content in muscle.

#### Muscle Shear force

A total of 56 microRNAs were DE between high and low shear force groups, of them 46 microRNA were downregulated and 10 microRNAs were upregulated in fish with high shear force (Fig. 2 and Supplementary Dataset 1C). Correlation between expression level and variation in muscle shear force was studied for 10 DE microRNAs in 90 fish, of them, 5 microRNAs (mir-126-3p, mir-148-3p*, mir-20-5p, mir-206 and mir-133a-5p) explained 6.5% to 9.0% variation in muscle shear force depending on the microRNA (FDR-p < 0.05) (Table 3). To the best of our knowledge, association of microRNAs with muscle firmness has not been previously reported in salmonids. The connective tissue proteins, especially collagen, play an important role in determining muscle shear force. Density of collagen fibers in connective tissue determines fish muscle firmness^36^. Interestingly, out of 56 DE microRNA, 31 microRNAs targeted 49 genes coding for collagen proteins or regulators of collagen biosynthesis, metabolism or structure (Supplementary Dataset 2, see name and GO terms of target genes). In addition, 31 DE microRNAs targeted at least 61 genes coding for extracellular matrix proteins and their regulators other than collagens and collagen regulators (Supplementary Dataset 2, see GO terms of target genes). It has been observed that abundance of collagen and connective tissue in extracellular matrix determines stiffness and gaping in fish muscle^37^. These DE microRNAs, which target collagens and other extracellular matrix connective tissues and their regulators, may be used to develop suitable genetic markers to improve muscle firmness in rainbow trout.

#### Muscle whiteness index

Seventy-seven microRNAs were DE, 58 upregulated and 19 downregulated, in fish families with high muscle whiteness index compared to low whiteness index families (Fig. 2 and Supplementary Dataset 1C). From DE microRNA, correlation between microRNA expression level and variation in phenotype was performed for 11 microRNAs, of them, variation in expression level of 6 microRNAs significantly correlated with variation in muscle whiteness index (Table 3). Expression of mir-20-5p, mir-148-3p* and mir-206 explained 8.8%, 8.7% and 8.4% of the variation in muscle whiteness respectively. Gene enrichment analysis of DE microRNA targets revealed that 30 biological pathways were significantly overrepresented which included transcription, transcription regulation, multicellular organism development, protein ubiquitination and Wnt signaling (Supplementary Dataset 4). We observed that most of the DE microRNA in response to variation in muscle yield, crude fat content and shear force were also DE in response to variation in muscle whiteness. These data suggest that muscle whiteness may be, at least partially, impacted by the mechanisms that regulate the other three muscle quality traits. Muscle color parameters correlate to muscle fat content in Atlantic salmon^38^. Perhaps this is among the first genome-wide studies aimed at exploring the genetic/molecular basis of muscle whiteness in salmonids.

### MicroRNA-target gene co-expression and transcriptional regulation

MicroRNAs and their target genes are usually co-expressed and co-regulated by common transcription factors^39^. To investigate co-expression of DE microRNAs and their target genes, first, we calculated Pearson correlation between each DE microRNA and their target genes based on their expression values in 30 RNA-Seq samples (see methods section). Next, we investigated whether this co-expression was regulated via common transcription factors (TF). For this purpose, we scanned promoter sequences of the strongly correlated (R^2^ > 0.70) DE microRNAs and their target genes for TF binding cis regulatory motifs. Out of 90 DE microRNAs, 15 microRNAs had strong positive expression correlation (R^2^ > 0.70) with 194 different target genes, and these correlated microRNA-target gene pairs shared common TF binding motifs in their promoters. Selected microRNA-target gene pairs and common TF binding motifs in their promoters are given in Table 4. Some of those TFs are known to be heavily involved in muscle development (e. g. myoD, c-Fos, c-Jun, MAZ, NF-AT1, Smad3, Elk1, PEA3, NFI/CTF and NFY), development (e.g. HOXD9, HOXD10 and COE1), metabolism (e. g. HNF-3) and adipogenesis (e. g. C/EBPbeta). These findings suggest that myogenic TFs may regulate expression of muscle related microRNAs and their target genes. Wang and coworkers suggested that both miRNAs and TFs must stay active to simultaneously regulate their target genes^39^.

**Table 4:** Cis-regulatory transcription factor binding motifs that exist in promoter sequences of differentially expressed (DE) microRNAs and their positively correlated target genes.

MicroRNA	Target gene	MicroRNA-target gene expression correlation (R^2^)	Common cis regulatory promoter motifs in microRNA and target gene
mir-92-5p	GSONMT00020058001: homeobox protein orthopedia b-like	0.9	HNF-3beta, AML1, TFIID, HNF-3
mir-26a-5p	GSONMT00040924001: kelch repeat and btb domain-containing protein 11-like	0.9	AR, C/EBPbeta, c-Jun, Fli-1, IRF-2, NF-AT1, NF-AT2, NF-AT3, NF-muNR, POU1F1, PR B
let-7a-5p	GSONMT00042722001: inhibitor of growth protein 5-like	0.8	AP-3, AR, C/EBPbeta, CBF(2), CP2, GATA-1, HNF-3alpha, HNF-3beta, HOXA4, NF-Y, PR B, Smad3, SXR:RXR-alpha, TBP
mir-222-3p	GSONMT00046577001: synembryn-b-like isoform × 2	0.8	C/EBPgamma, c-Jun, NF-Y, RAR-gamma, Smad3, WT1 I
mir-10c-5p	GSONMT00022788001: transcription elongation factor SPT6-like	0.8	HNF-3alpha, Elk-1, HNF-3beta
let-7c-5p	GSONMT00013587001: collectrin-like isoform	0.8	AR, Crx, HNF-3alpha, HNF-3beta, HOXD10, HOXD9, Pax-2.1, PR B, TFIID
mir-130-5p	GSONMT00036000001: uncharacterized protein	0.8	CP2, HNF-3alpha, HNF-3beta, PR B, RAR-gamma
mir-27b-5p	GSONMT00073742001: serine threonine-protein phosphatase 6 regulatory ankyrin repeat subunit c	0.8	AhR, AR, c-Myb, NFI/CTF, POU1F1b, POU1F1c, PR A, PR B
mir-29c-5p	GSONMT00037095001: protein NLRC3-like	0.8	AP-3, ENKTF-1, HNF-3beta, NF-Y, PPAR-alpha:RXR-alpha, PR B
mir-221-5p	GSONMT00080720001: lathosterol oxidase	0.7	AR, C/EBPbeta, c-Jun, HNF-3beta, p53, PEA3, PR B
mir-132-3p	GSONMT00033564001: dihydropyrimidinase	0.7	AP-3, CBF(2), CP2, Elk-1, ER-alpha, ER-beta, MyoD, NF-1, NF-Y, PU.1, SF-1, T3R-beta
mir-30d-5p	GSONMT00002523001: solute carrier family 12 member 9-like	0.7	AR, PR B, CP2
mir-462-5p	GSONMT00075887001: ATP-dependent 6-phosphofructokinase, muscle type-like	0.7	HNF-3alpha, MyoD, NF-AT3, NF-AT2, PR B
mir-133a-3p	GSONMT00002111001: TPA_inf: tachykinin 4	0.7	c-Fos, c-Jun, COE1, GATA-1, HNF-3, HNF-3beta, IRF-1, MAZ, NF-AT1, Pbx1b, PEA3, PR B

### Genetic polymorphism in microRNA target sites

In order to explore phenotype-associated genetic polymorphism on microRNA and microRNA binding sites in target genes, first we predicted target genes of all rainbow trout microRNAs that were reported previously^18^. We performed SNP prediction on the mature microRNA sequences. In addition, SNPs on microRNA target genes were predicted by mRNA sequencing of the same fish families used in this study (data under review for publication elsewhere). A total of 249 SNPs existing in microRNA recognition element seed site (MRESS) of target genes showed allelic imbalance (>2.0 as an amplification and <0.5 as loss of heterozygosity) between high and low ranked families of different muscle traits. Out of 249 SNPs, 240 SNPs either destroyed or created a novel illegitimate microRNA target, and only 9 SNPs had no potential effect on microRNA binding (Supplementary Dataset 5). This alteration in target recognition is due to presence of the SNPs in MRESS which plays crucial role in microRNA-mRNA binding^1^. Any SNP in the MRESS has an important impact on microRNA recognition^7^. Out of 240 SNPs capable of destroying or creating a novel illegitimate MRESS, 204 SNPs were found to be true polymorphic SNPs by genotyping fish, and showed allelic imbalance between high and low ranked families (>2.0 as an amplification and <0.5 as loss of heterozygosity). Interestingly, 78 unique SNPs showed significant association with growth and muscle quality phenotypes based on genotype-phenotype association analysis performed on a larger number of fish from 2 generations (n = 786) (FDR-p-value < 0.05). Table 5 lists selected SNPs with high correlation coefficients with growth traits while complete list of SNPs in microRNA binding sites and their association with phenotype is given in Supplementary Dataset 5. A total of 55, 28, 7 and 9 MRESS-destroying SNPs in 3′ UTR were significantly associated with WBW, muscle yield, crude fat content and muscle whiteness respectively (FDR-p-value < 0.05).

**Table 5:** SNPs in microRNA recognition element seed site (MRESS) of target gene, allele frequency ratio of MRESS-destroying SNPs between high vs low ranked families of different muscle traits, and correlation between the SNP and phenotype.

MicroRNA	MRESS with SNP	SNP NCBI Sr.	MRESS destroying allele	Frequency ratio of MRESS destroying allele (High/Low family) (WBW; MY; CFC; SF; MWI)	Target gene harboring SNP	SNP association with phenotype (R^2^) (WBW; MY; CFC; SF; MWI)
pma-miR-7a-3p	TGTC[C/T]TGT	2711239550	T	11.00; 3.55; 2.33; 0.96; 1.21	troponin fast skeletal muscle isoform	0.042; NA; NA; NA; NA
cgr-miR-598	TCCTAC[T/G]A	2711263652	G	0.30; 0.44; 0.11; 0.25; 0.31	malate dehydrogenase cytoplasmic-like	0.031; NA; NA; NA; 0.019
efu-miR-9203a	ACTATC[C/T]AA	2711277723	T	1.62; 1.04; 3.00; 0.83; 1.00	ranbp-type and c3hc4-type zinc finger-containing protein 1	0.031; NA; NA; NA; NA
oha-miR-30e-5p	AC[C/T]GGAAGG	2711281551	C	3.24; 5.00; 1.06; 0.27; 0.25	phosphate carrier mitochondrial precursor	0.026; NA; NA; NA; 0.019
ssa-miR-139-5p	CA[C/A]TGTAGA	2711237958	A	0.29; 1.57; 0.37; 0/0.25; 0.39	novel protein vertebrate nebulin	0.028; NA; NA; NA; NA
ssa-miR-139-5p	CACT[G/A]TAGA	2711228680	A	0.29; 1.57; 0.37; 0/0.25; 0.39	novel protein vertebrate nebulin	0.028; NA; NA; NA; NA
dvi-miR-968-5p	TATCAT[C/T]AG	2711283561	C	5.50; 1.00; 0.56; 30.67; 2.57	ATP-dependent 6-phosphofructokinase, muscle type	0.026; NA; NA; NA; NA
ssy-miR-508	GT[A/G]GCTGG	2711210896	A	2.56; 0.68; 9.20; 2.52; 0.58	ankyrin repeat and socs box protein 5	0.029; 0.016; NA; NA; NA
ppc-miR-8229a-5p	GCTGAGG[A/T]	2711244497	T	5.09; 5.48; 4.00; 0.18; 1.17	calsequestrin-1-like	0.021; NA; NA; NA; 0.012
oha-miR-26-5p	GGATA[C/A]GGT	2711271866	A	1.11; 1.71; 1.40; 0.16; 0.25	fk506-binding protein 1a	0.027; 0.015; NA; NA; NA
oha-miR-24-3p	AGCAG[G/A]AAA	2711198793	A	1.83; 0.35; 0.44; 1.90; 2.20	spectrin beta non-erythrocytic 1 isoform ×1	0.019; NA; NA; NA; NA
mmu-miR-3547-3p	CCCC[T/C]CTT	2711211990	C	0.31; 3.21; 0.20; 1.00; 0.27	gamma-adducin-like isoform ×6	0.024; 0.031; NA; NA; NA
oha-miR-365a-2-5p	CAGAA[G/A]GA	2711268945	A	2.16; 0.55; 8.44; 0.43; 2.50	nuclear receptor subfamily 1 group d member 2	0.020; NA; NA; NA; NA
ssa-miR-196b-5p	GT[A/T]GTTGTT	2711240153	A	0.40; 0.09; 1.50; 0.53; 0.64	histone-lysine n-methyltransferase setd7-like	0.024; NA; NA; NA; NA
ppc-miR-8298-3p	CATA[A/C]TTC	2711225442	A	0.67; 1.81; 0.50; 0.39; 0.51	atp synthase lipid-binding mitochondrial precursor	0.024; 0.024; NA; NA; NA
oha-miR-181a-5p	T[G/T]AATGTT	2711263991	T	3.75; 1.89; 1.21; 2.13; 0.67	phosducin-like protein 3	0.020; NA; NA; NA; NA
eca-miR-9171	CAG[A/G]CTGT	2711198125	G	2.10; 1.00; 0.56; 1.40; 6.50	nfu1 iron-sulfur cluster scaffold mitochondrial-like	0.022; NA; NA; NA; NA
mmu-miR-7241-3p	AGTATT[C/T]G	2711278279	T	1.75; 5.50; 0.42; 1.25; 0.60	3-hydroxyacyl-CoA dehydratase 1	0.017; 0.018; NA; NA; NA
cgr-miR-29a-5p	GTGTACG[G/T]C	2711192504	G	2.89; 1.00; 11.33; 3.33; 1.47	phosphorylase b kinase gamma catalytic subunit	0.015; 0.017; NA; NA; NA
efu-miR-9186e	TCT[C/A]TGGTA	2711207216	C	2.05; 3.08; 21.11; 0.37; 2.16	manganese superoxide dismutase	0.019; 0.013; NA; NA; NA
ccr-miR-729	TACCCA[T/C]C	2711280997	C	5.47; 2.86; 2.75; 21.11; 0.40	profilin-2-like isoform ×1	0.016; NA; NA; NA; NA
cfa-miR-8804	TCTA[T/C]CTA	2711247534	C	0.64; 0.32; 0.11; 0.43; 0.79	14-3-3 protein beta alpha-2	0.017; 0.019; NA; NA; NA
ssa-miR-210-5p	[T/G]TACATTA	2711275155	T	3.26; 0.49; 1.07; 0.90; 6.51	60 s ribosomal protein l17	0.016; 0.024; NA; NA; NA
eca-miR-9011	[T/A]CCTGTACA	2711195787	T	0.29; 8.80; 0.90; 0.44; 1.14	monocarboxylate transporter 9	0.010; NA; NA; NA; NA
api-miR-3015a	TTGAAAAC[C/A]	2711235305	C	1.32; 2.67; 3.78; 0.58; 0.82	parvalbumin-7-like isoform ×2	0.015; NA; NA; NA; 0.012
ssa-miR-93a-5p	AGCA[T/C]TTTG	2711192683	T	0.99; 0.72; 7.82; 0.25; 5.09	protein nap homolog 2-like	0.013; NA; 0.02; NA; 0.02
mmu-miR-6975-3p	[G/A]CAGACGAC	2711260618	G	0.46; 0.08; 2.53; 0.98; 6.33	wolframin	0.012; 0.012; NA; NA; NA
hsa-miR-8086	ATGTAT[T/G]CC	2711280737	T	0.08; 0.17; 0.74; 0.66; 0.19	muscleblind-like protein 1-like	NA; 0.010; NA; NA; NA
osa-miR818f	ACAATCT[A/G]T	2711274522	G	0.21; 0.13; 0.40; 5.44; 1.68	nexilin isoform ×1	NA; 0.012; NA; NA; NA
mmu-miR-6989	GCAACT[C/T]CAA	2711191913	T	0.21; 0.11; 0.74; 2.70; 1.00	arrestin domain-containing protein 2	NA; 0.013; NA; NA; NA
ggo-miR-4738	AGCAGC[G/A]T	2711275288	G	4.11; 12.50; 2.48; 0.18; 0.71	sarcosine mitochondrial-like	NA; 0.013; NA; NA; NA
prd-miR-7957d-3p	TC[T/A]GGACAT	2711274238	T	4.74; 15.63; 2.48; 0.18; 0.60	profilin-2-like isoform ×2	NA; 0.013; NA; NA; NA
oha-miR-133b-5p	GTGCAC[G/T]T	2711249814	G	0.09; 0.18; 0.25; 38.00; 0.85	inactive dual specificity phosphatase 27	NA; 0.016; NA; NA; NA
ppc-miR-8304a-3p	[C/A]TTTGG	2711194746	A	0.17; 2.71; 0.14; 1.90; 0.50	atlastin-2 isoform 1	NA; NA; 0.017; NA; NA
ipu-miR-34b	TGTT[A/C]ACT	2711267634	C	1.64; 2.87; 3.71; 0.50; 1.41	creatine kinase m-type-like	NA; NA; 0.019; NA; NA

Column 1 shows potential regulatory microRNA and column 2 shows microRNA binding site in 3′ UTR of target gene. Note that only MRESS with SNP, not full microRNA sequence is shown. Complete datasets are given in Supplementary Dataset 5. Note: WBW: whole body weight, MY: muscle yield of WBW (%), CF: muscle crude fat content (%), SF: muscle shear force; and MWI: muscle whiteness index.

Previous reports indicated that SNPs in MRESS of target genes have important impact in phenotype determination^7^. Phenotype associated MRESS-destroying SNPs were present in various classes of genes including metabolic enzymes, transporter proteins, transcription factors, signaling molecules and muscle related proteins. Among the WBW associated SNPs, those present in 3′ UTR of troponin, malate dehydrogenase, ranbp-type and c3hc4-type zinc finger-containing protein 1, phosphate carrier mitochondrial like protein, cytochrome b-c1 complex, novel protein vertebrate nebulin, ATP-dependent 6-phosphofructokinase, ankyrin repeat and socs box protein 5 and calsequestrin-1 each SNP explained more than 2% variation in WBW phenotype (Table 5 and Supplementary Dataset 5). Similarly, SNPs existing in 3′ UTR of beta-sarcoglycan, 60 S ribosomal protein 117, ATP-synthase lipid binding protein and gamma-adducin-like isoform × 6 each explained over 2% variation in muscle yield. Presence of MRESS-destroying alleles may stabilize the target genes against microRNA-mediated downregulation, which may contribute to the difference in phenotype between high vs low ranked families. As an example, allele destroying MRESS in troponin fast skeletal muscle was 11 times more frequent in high WBW family compared to low WBW family, and explained 4.2% variation in WBW (Table 5). Previous study performed in this trout population has also identified 3 SNPs present in 3′ UTR of troponin C associated with growth traits in trout^40^. Similarly, allele destroying MRESS in ATP-dependent 6-phosphofructokinase, a glycolytic gene, was 5.5 times more frequent in high WBW family compared to low WBW family, and explained 2.6% variation in WBW. Consistent with our finding, SNPs associated with growth traits in trout from our previous study were mainly present in genes involved in energy metabolism and muscle structure^40^. Above findings suggest that 3′ UTR SNPs capable of destroying or creating an illegitimate MRESS may have important functional consequences.

In contrast to target genes, no SNPs with allelic imbalances were detected in the microRNA mature sequences. This may be due to high degree of negative selective pressure as hundreds of genes are regulated by the same microRNA^3^. Previous studies from mammals suggest that SNPs in microRNA sequence, especially seed region, are rare due to high degree of selective pressure^41^. These findings suggest that due to high selective pressure to conserve microRNA sequence, cell may introduce genetic variation in microRNA binding site of appropriate target gene to regulate the phenotype.

## Conclusion

Improvement of muscle growth and quality in salmonids has long been sought by aquaculture industries. So far, little progress has been made toward genetic improvement of muscle growth and quality in salmonids. Muscle quality traits are lethally-measured traits which are hard to include in a breeding objective. In addition, complex interrelated relationship among muscle quality traits^19, 42^, and influence of several genetic and non-genetic factors complicates genomic selection. Previous studies have found genetic variation for muscle traits in trout and other salmonids^43–45^. Genomic technologies could be used to exploit within-family genetic variation for these lethally-measured muscle quality traits to identify suitable genetic markers. In this study, we investigated microRNA expression and genetic polymorphism in microRNA-binding sites in target genes associated with phenotypic variation in muscle growth and quality in a pedigreed rainbow trout population undergoing selection for improved growth performance.

Muscle specific myogenic microRNAs (e. g. mir-1, mir-206 and mir-133) as well as several non-muscle specific microRNAs showed regulated expression in response to variation in muscle yield and other muscle quality phenotypes. Most of the DE microRNAs between high vs low muscle yield families were also DE between high vs low families of muscle whiteness and muscle shear force phenotype. This observation may be, in part, due to interrelated nature of these muscle growth phenotypes^19, 42^. Biological pathways such as development, cell cycle, muscle growth, transcription and muscle proteolysis were significantly overrepresented in the list of DE microRNA targets suggesting that DE microRNAs may regulate growth and muscle quality traits by post-transcriptionally regulating the genes involved in these pathways. Presence of common cis regulatory motifs for myogenic TFs in DE microRNAs and their respective target genes may suggest that myogenic TFs may regulate expression of both myogenic microRNAs and their target genes.

Due to crucial role of MRESS in microRNA-mediated gene regulation, SNPs creating or destroying MRESS in target genes have important functional consequences^7, 46^. In this study, we identified 72 true SNPs in 3′ UTR capable of abrogating or creating MRESS on several metabolic and growth-related genes, which explained significant variation in growth, muscle yield and other muscle phenotypes. However, functional significance of these SNPs in microRNA target recognition needs to be experimentally validated. To make the present study more robust in identifying potential genetic markers for muscle and growth phenotypes, we applied dual approach by analyzing differential microRNA expression as well as genetic variation analysis in their target genes. We believe that this approach is more productive as high negative selective pressure imposes constraints on expression/genetic variation in microRNA^41^. In support of this argument, we found several SNPs on microRNA binding site of target genes that showed allelic-imbalance between high and low WBW groups though microRNA itself did not show variation in sequence or expression between the two groups. In this study, we performed a genome wide approach to investigate variation in expression and variation in target recognition of growth and muscle-important microRNAs, and the study may help identify suitable genetic marker for genetic selection of these traits.

In a separate study performed in the same set of fish, an unexpectedly low number of protein coding genes were observed as DE between high and low ranked families of these phenotypes (data will be published elsewhere). But, in this study, we observed significant number of DE microRNAs and their target protein coding genes that are associated with these phenotypes. These findings suggest that variation in these muscle quality phenotypes may be explained largely by variation in microRNA expression and genetic variation affecting recognition of microRNA targets rather than by direct regulation of mRNA expression. Consequently, the current study pinpoints to a greater role of microRNA-mediated gene regulation in determination of muscle traits in rainbow trout.

Using genomics approach, we have identified significant correlation of microRNA gene expression and SNPs in microRNA binding sites with growth/muscle quality traits of economic importance. Some of the genotype x phenotype associations reported in this study although significant, present low-to-medium values, not allowing to make definitive inferences about the behavior of some characteristics of the muscle growth and quality traits. The highly polygenic nature of traits demands identification of more genetic markers. Incorporation of genomics selection, based on genetic markers, can greatly help traditional breeding programs, based on quantitative evaluation techniques, in improving accuracy of breeding value prediction.

## Materials and Methods

### Ethics statement

Fish were maintained at the NCCCWA and all experimental protocols and animal procedures were approved and carried out in accordance with the guidelines of NCCCWA Institutional Animal Care and Use Committee Protocols #053 and #076.

### Fish population and muscle sampling

Phenotypic data and muscle samples were collected from 471 fish representing 98 families (~5 fish/family) from USDA/NCCCWA growth selected trout line from each harvest year 2010 as previously described^40^. Briefly, fish families were produced and reared till ~13 month post-hatch as previously described^17^. Single-sire x single-dam mating was used to produce full sib families and eggs were reared in spring water. Water temperature was manipulated from 7 °C to 13 °C to synchronize the hatching time. Each family was reared in a separate 200-L tank at ~600 alevins/tank density. Random culling of fish was performed every month to maintain stock density of <50 kg/m^3^. At the age of 5 month, each fish was given unique identification PIT (passive integrated transponder) tag, and tagged fish were combined and reared in a big 1,000-L commercial tanks. Commercial fishmeal-based diet (16% fat, 42% protein; Ziegler Bros Inc., Gardners, PA) was fed using automatic feeder. Feeding rate was gradually reduced from 2.5% of body weight to 0.5% of body weight as fish grew older. WBW of all fish belonging to 98 families was measured, and families were ranked based on their WBW measurements. Second or third ranked fish from each family was selected for muscle sampling so that WBW of sampled fish is adjusted around median of each family. Selected fish were randomly assigned to one of the 5 harvest group (~100 fish/harvest group) and each harvest groups were sampled in 5 consecutive weeks. Fish were anesthetized in 100 mg/L tricaine methanesulfonate and weighted, slaughtered and eviscerated. Muscle samples were separated from dorsal musculature and were stored in liquid nitrogen until processing and phenotype measurement. Muscle quality phenotypes were measured at West Virginia University (WVU), meat processing laboratory. Muscle yield was calculated as percentage of WBW, and proximate analysis of muscle fillet was performed according to previously described protocol^47^. Briefly, fresh fillet surface color was measured with a chromameter (Minolta, Model CR-300; Minolta Camera Co., Osaka, Japan) calibrated using a standard white plate No. 21333180 (CIE Y 93.1; ×0.3161; y 0.3326). L* (lightness), a* (redness), and b* (yellowness) values were recorded at three locations above the lateral line along the long axis of the right fillet, and these values were used to calculate a fillet whiteness index according to the following equation; whiteness index = 100 − [(100 − L)^2^ + a^2^ + b^2^]^1/2^ ^48^. Muscle crude fat content was measured using Soxhlet solvent extractor with petroleum ether. For muscle peak shear force measurement, texture was analyzed using a five blade Allo-Kramer shear attached to the texture analyzer; and Texture Expert Exceed software (version 2.60; Stable Micro Systems Ltd., Surrey, U.K.) was used to record the peak shear force.

### Library construction and sequencing

For differential gene expression analysis, 4 highest ranked and four lowest ranked families for each trait were sequenced. White muscle sample was isolated from five individuals belonging to each family and total RNA was isolated using Trizol protocol (Invitrogen, Carlsbad, CA) as described previously^49, 50^. To allow enough transcriptome sequence total RNA from 5 individual fish from each family was pooled and sequenced on Illumina’s HiSeq platform (Illumina Inc, CA, USA).

### Data processing and prediction of trout microRNA

Sequencing adapter 5′GCCTTGGCACCCGAGAATTCCA-3′ was trimmed and reads were annotated using miRBase microRNA reference (release 21) in CLC Bio small RNA analysis tool. Read alignment was run at default settings (i.e. mismatch ≤ 2, additional/missing upstream/downstream bases ≤ 2). MicroRNAs with mismatches and/or additional/missing upstream/downstream nucleotides were considered as variants of the same microRNA. Read count from all variants of the same microRNAs were summed and were used as expression value for that particular microRNA (default method of merging expression values in CLC Bio’s small RNA analysis toolkit).

### Identification of DE microRNAs

DE microRNAs were identified using EDGE test in CLC genomics workbench using expression values from the above step. The fold change in gene expression between two groups was considered significant if FDR-p < 0.05 and fold change <−2 or >2 fold.

### Real time PCR validation of DE microRNAs and ‘microRNA expression-phenotype variation’ correlation

Same RNA samples from high and low ranked families used for sequencing were used to validate RNA-Seq differential expression. cDNA was synthesized using miScript II RT kit (Qiagen, Valencia, CA, USA) and microRNA was quantified in Bio-Rad CFX96™ Real Time System (Bio-Rad, Hercules, CA) using miScript^R^ SYBR^R^ green (Qiagen, Valencia, CA, USA). A non-coding RNA U6 was used as a endogenous control for normalization, and fold change was calculated by ΔΔCt method^51^, as described previously^52, 53^.

Correlation between microRNA expression and phenotype was studied in 90 random individual fish from 3^rd^-generation families (2010 hatch year) from the growth-selected line. ΔCt value of each microRNA in all 90 fish was calculated. Pearson correlation and simple linear regression was run between ΔCt and quantitative value of muscle phenotype.

### Bioinformatics prediction of DE microRNA target

For prediction of microRNA targets, 3′-UTR of trout mRNA were retrieved from the genome reference^54^. Due to difference in sensitivity and specificity of different target prediction algorithms, targets were predicted using 3 tools: miRanda, PITA and TargetSpy in small RNA analysis server sRNAtoolbox^55^. If the same target site is predicted by all 3 tools it was considered a potential microRNA target. For PITA, prediction parameters chosen were: seed length 6–8 nts, no G:U wobble allowed in seed of size 6 nts, one G:U wobble allowed in seed of size 7–8 nts, no mismatches allowed in seed of size 6 and 7 nts, one mismatch allowed in seed of size 8 nts, and no loop is allowed in microRNA or target for any seed size. Miranda parameters chosen for target prediction were: score threshold 150, gap-open penalty −4.0 and gap-extend penalty 9.0. For TargetSpy score a minimum threshold of 0.99 was used. For all tools, minimum energy threshold was chosen as −15Kcal/mole.

### Gene enrichment analysis of microRNA targets

Gene enrichment analysis (GEA) was performed by using DAVID ^20, 21^ (FDR-p < 0.05) and overrepresented pathways were visualized by EnrichmentMap app^56^ in cytoscape^57^ (p value < 0.005 and FDR-q < 0.05). Overlap between gene-sets was computed according to overlap coefficient, which was set to the default recommended value of 0.5.

### MicroRNA-target gene co-expression and identification of cis regulatory promoter motifs

Small RNA and mRNA sequencing reads from 22 families were used to calculate microRNA-target gene correlation. For target genes, mRNA sequencing reads were mapped to trout mRNA reference and transcript per million (TPM) was calculated for each mRNA. For microRNA, small RNA sequencing reads were mapped to mature microRNA sequence from miRBase release 21 (June 2014) and total count was calculated for each microRNA. TPM (for mRNA) and total count (for microRNA) were normalized and used to calculate expression correlation.

Transcription factor (TF) binding motifs were searched in the 500 upstream promoter sequences of DE microRNAs and correlated target genes using.Alggen Promo TF motif search tool^58, 59^. Search parameters used were ‘only teleost transcription factors’ and ‘only teleost motif sites’. Maximum dissimilarity rate between putative and consensus TF binding site was set to 5%, and RE equality/query (expectation of finding motif in random sequence) was set to <0.05.

### Prediction of single nucleotide polymorphism (SNP)

The same RNA samples used to sequence small RNAs were used to sequence protein coding genes. Details of SNP prediction are described in a publication under review. Briefly, sequencing libraries were prepared using Illumina’s Truseq RNA library preparation kit and sequenced on Illumina’s HiSeq platform (Illumina Inc, CA, USA). GATK^60^ and SAMTool^61^ were both used to call SNPs. In the SAMtool approach, STAR alignment tool^62^ was used to align sequencing reads from each family to the trout genome. SAMtools view/sort and mpileup functions were used to determine variant genotype. Popoolation2 package (version 1.201) was used to calculate allele frequencies^61, 63^. Initial SNPs were considered at minimum reads >10 and minor allele count >4 and minimum allelic frequency (MAF) >0.05. SNPs were considered as putative trait-associated SNPs if allele frequency (allele A/allele B) ratio between high and low ranked families was ≥2.0 (as an amplification) or ≤0.5 (as loss of heterozygosity). In GATK approach, reads were aligned to the genome using STAR alignment tool and then Picard tool was used to sort the SAM files and to mark duplicates. Split and trim function was performed to reassign mapping quality, Indel realignment and local realignment around the Indel to clean up any mapping artifacts. Finally, base quality score recalibration was performed on the data resulting from Picard tools. Haplotype Caller was used to determine variants, followed by filtration of SNPs using strict thresholds: Qual By Depth (QD) 2.0, Fisher Strand (FS) 60.0, RMS Mapping Quality (MQ) 40.0 and MAF > 0.05.

### SNP genotyping and SNP-phenotype association analysis

SNP genotyping was performed as a part of development of a 50 K SNP chip for rainbow trout (full SNP chip study will be published elsewhere). Putative SNPs were genotyped in ~1,900 individuals from USDA, NCCCWA growth selected lines harvest year 2010 and 2012 using Affymetrix SNP array protocol (Geneseek Inc., Lincoln, NE, USA). Briefly, genomic DNA from each fish was extracted from a fin clip and was amplified by PCR. DNA samples were chemically fragmented and then biotinylated. Biotinylated DNA samples were hybridized to DNA probe in the SNP array and genotype call was determined based on ‘probe DNA: sample DNA’ hybridization.

For SSNP-phenotype association study, only 249 SNPs present in 3′ UTR microRNA binding sites and only 786 individual fish from 3^rd^- and 4^th^-generation families (2010 and 2012 hatch years)^17^ with available phenotype measurements of interest were considered. Phenotypic data were rank transformed^64^, then data normality was checked by Kolmogorov-Smirnov and Shapiro-Wilk test to make sure that the traits are normally distributed to meet the assumption of quantitative trait association analyses. SNP-phenotype association was performed by quantitative trait analysis, linear and logistic methods in PLINK tool^65^ and all three methods gave consistent results, and results from quantitative trait analysis were reported. Quantitative association analysis was performed between SNP genotype and quantitative value of phenotype measurements of 786 individuals. Linear association between SNP genotypes and phenotype measurement was performed to get correlation coefficient (R^2^) between the SNP allele and the phenotype. Logistic regression was performed to check if the effect of one SNP on the phenotype was independent of other SNPs. Associations of all reported SNPs were significant in linear and quantitative analysis after entering other SNPs as covariates in logistic method, suggesting that the effect of each SNP for association to the phenotype is independent from other SNPs. Population stratification analysis was performed using genome-wide IBS that is integrated in PLINK. Multidimensional scaling plot on N x N matrix of genome-wide IBS pairwise distances showed fairly homogeneous sample with no obvious population stratification or significan clustering (Supplementary Dataset 1E).

## Electronic supplementary material

Supplementary Dataset 1

Supplementary Dataset 2

Supplementary Dataset 3

Supplementary Dataset 4

Supplementary Dataset 5
