# Supplementary material for: RNA-Seq reveals MicroRNA expression signature and genetic polymorphism associated with growth and muscle quality traits in rainbow trout
Source: Sci Rep. 2017 Aug 22;7:9078. doi: 10.1038/s41598-017-09515-4 (PMC5567286; doi:10.1038/s41598-017-09515-4)
Supplement: Supplementary file 1 — Supplementary Dataset 1 [file 41598_2017_9515_MOESM1_ESM.doc]

RNA-Seq reveals MicroRNA expression signature and genetic polymorphism associated with growth and muscle quality traits in rainbow trout

Bam Dev Paneru^1^, Rafet Al-Tobasei^2^, Brett Kenney^3^, Timothy D. Leeds^4^ and Mohamed Salem^1,2*^

^1^Department of Biology and Molecular Biosciences Program, Middle Tennessee State University, Murfreesboro, TN, 37132, U.S.

^2^Computational Science Program, Middle Tennessee State University, Murfreesboro, TN 37132, U.S.

^3^Division of Animal and Nutritional Science, West Virginia University, Morgantown, 26506‐6108, West Virginia

^4^The National Center for Cool and Cold Water Aquaculture, USDA Agricultural Research Service, Kearneysville, WV 25430, U.S.

*Correspondence:

Mohamed Salem

Department of Biology and Molecular Biosciences Program,

Middle Tennessee State University,

Murfreesboro, TN, 37132, U.S.

Mohamed.salem@mtsu.edu

**Supplementary Dataset 1A**: Read length distribution of small RNA sequencing reads before and after sequencing adapter trimming. Note that after trimming of sequencing adapter, average read length is 22 nucleotides, which is a typical length for most of the characterized mature microRNAs.


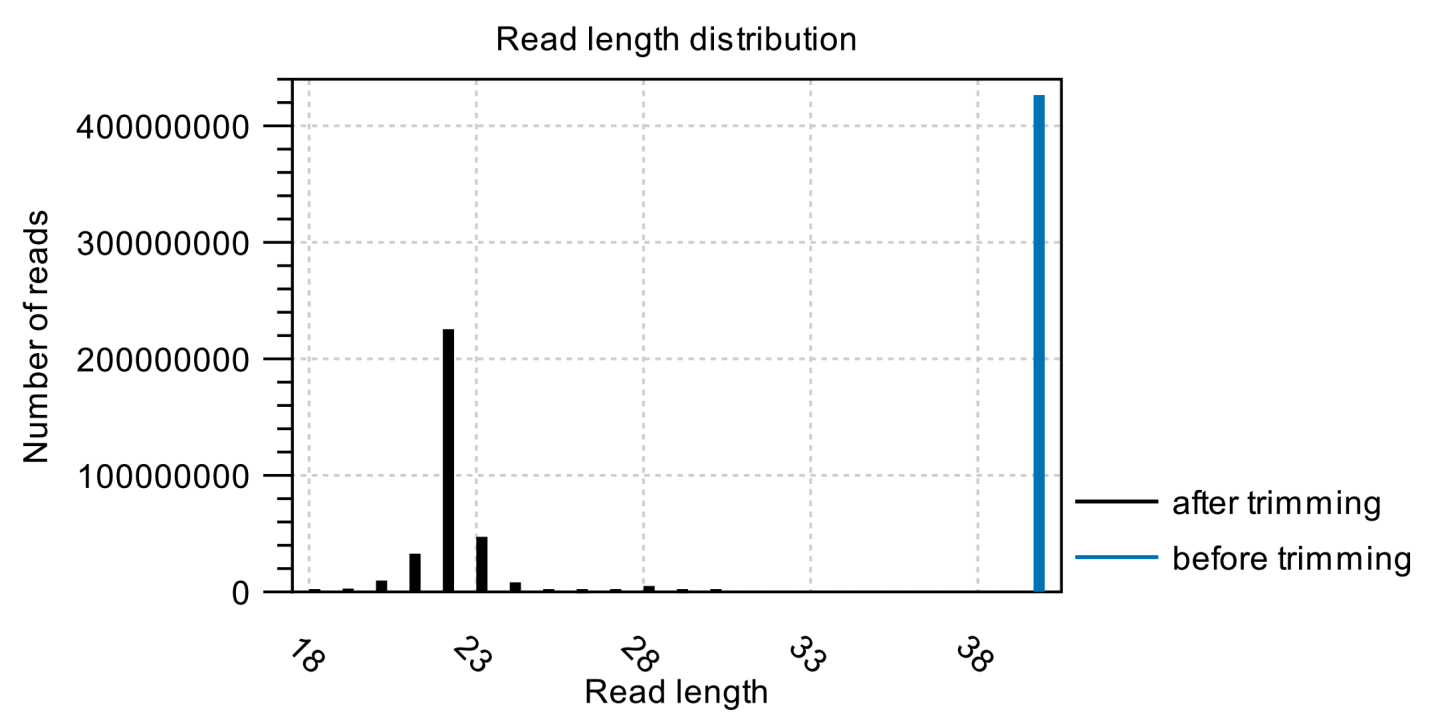


**Supplementary Dataset 1A**: RNA seq principal component analysis (PCA) of 22 fish families used in the study. PCA scatter plot shows no clustering of fish families.


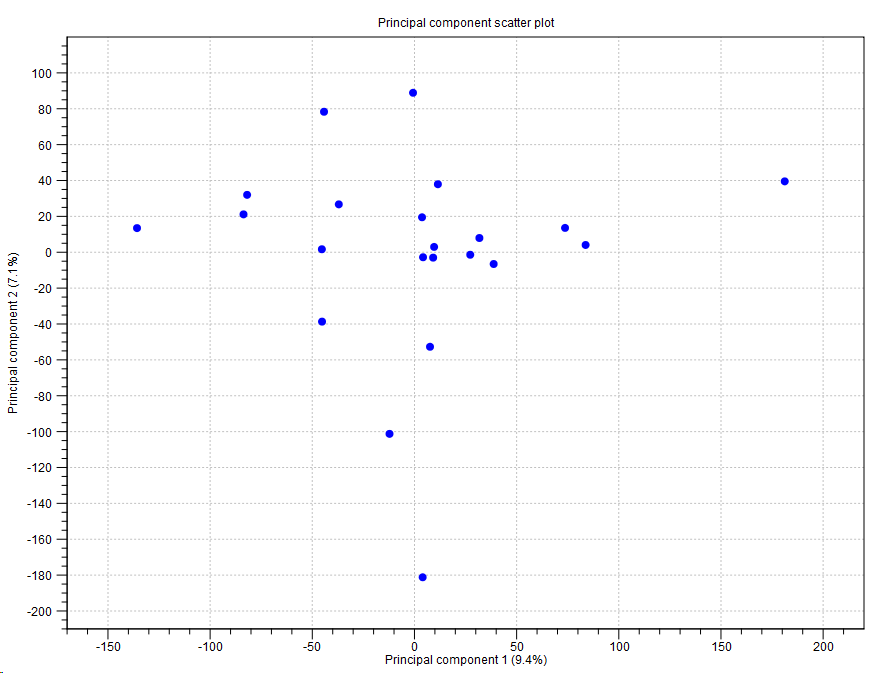


**Supplementary Dataset 1C**: Differentially expressed microRNAs between high and low ranked families of different muscle traits.

| **MicroRNA name** | **MicroRNA sequence** | **Fold change in high ranked families relative to low ranked families for each trait** | | | | | | | |
| --- | --- | --- | --- | --- | --- | --- | --- | --- | --- |
|  |  | **Muscle yield** | | **Crude fat content** | | **Shear force of muscle** | | **Fillet whiteness** | |
|  |  | **Fold change** | **FDR p-value correction** | **Fold change** | **Fold change** | **FDR p-value correction** | **FDR p-value correction** | **Fold change** | **FDR p-value correction** |
| mir-1-5p | ATACATACTTCTTTACATTCCA | -145.4 | 0.0010 | -173.4 | 168.5 | <0.00001 | 0.0010 | NA | NA |
| mir-143-5p | GGTGCAGTGCTGCATCTCTGGT | -110.9 | 0.0078 | -11.1 | 11.3 | 0.0001 | 0.0078 | NA | NA |
|  |  |  |  |  |  |  |  |  |  |
| mir-1a-3p | TGGAATGTAAAGAAGTATGTAT | -83.9 | 0.0012 | -93.2 | 98.5 | <0.00001 | 0.0012 | NA | NA |
| mir-126-3p | TCGTACCGTGAGTAATAATGCA | -60.1 | 0.0012 | -7.8 | 12.6 | <0.00001 | 0.0012 | NA | NA |
| mir-19d-5p | TGTGCAAACCCATGCAAAACTGA | -48.8 | 0.0012 | -50.4 | -6.3 | 0.0137 | 0.0012 | NA | NA |
| mir-140-3p | TACCACAGGGTAGAACCACGG | -35.1 | 0.0015 | -14.6 | 31.5 | <0.00001 | 0.0015 | NA | NA |
| mir-100-5p | AACCCGTAGATCCGAACTTGTG | -20.3 | 0.0071 | -23.6 | 3.3 | 0.0261 | 0.0071 | NA | NA |
| mir-99a-5p | AACCCGTAGATCCGATCTTGTG | -18.3 | 0.0239 | -22.0 | 5.4 | 0.0019 | 0.0239 | NA | NA |
| mir-148-3p | TCAGTGCATTACAGAACTTTAT | -18.1 | 0.0071 | -23.2 | 27.0 | <0.00001 | 0.0071 | NA | NA |
| mir-20-5p | TAAAGTGCTTATAGTGCAGGTAG | -17.4 | 0.0045 | -17.3 | 14.4 | <0.00001 | 0.0045 | NA | NA |
| mir-199-5p | CCCAGTGTTCAGACTACCTGTTC | -17.1 | 0.0432 | -31.9 | 5.1 | 0.0025 | 0.0432 | NA | NA |
| mir-148-3p* | TCAGTGCATAACAGAACTTTGG | -15.8 | 0.0080 | -28.2 | 21.4 | <0.00001 | 0.0080 | NA | NA |
| mir-206 | TGGAATGTAAGGAAGTGTGTGGT | -15.6 | 0.0071 | -12.6 | 12.0 | 0.0002 | 0.0071 | NA | NA |
| mir-16-5p | TAGCAGCACGTAAATATTGGAG | -14.0 | 0.0043 | -28.5 | 12.4 | 0.0004 | 0.0043 | NA | NA |
| mir-19b-3p | TGTGCAAATCCATGCAAAACTGA | -9.3 | 0.0166 | -8.7 | -7.5 | 0.0087 | 0.0166 | -14.1 | 0.0435 |
| mir-133a-5p | AGCTGGTAAAAAGGAACCAAATC | -8.9 | 0.0321 | -5.8 | 5.1 | 0.0043 | 0.0321 | NA | NA |
| mir-145-3p | GGATTCCTGGAAATACTGTTCT | -8.6 | 0.0449 | -16.8 | 18.1 | <0.00001 | 0.0449 | NA | NA |
| mir-140-3p* | CAGTGGTTTTACCCTATGGTAG | -8.4 | 0.0432 | -9.4 | 9.6 | 0.0003 | 0.0432 | NA | NA |
| mir-191 | CAACGGAATCCCAACAGCAG | NA | NA | NA | -101.2 | 0.0063 | NA | -951.9 | 0.0111 |
| mir-99b | CACCCGTAGAACCGGCCATGC | NA | NA | NA | -50.7 | 0.0005 | NA | -103.6 | 0.0435 |
| mir-10c-5p | TACCCTGTAGATCCGGATTTGT | NA | NA | 8.7 | -11.3 | 0.0005 | NA | -36.0 | 0.0111 |
| mir-10b-5p | TACCCTGTAGAACCGAATTTGT | NA | NA | 8.8 | -11.6 | 0.0018 | NA | -23.8 | 0.0111 |
| mir-301b-3p | CAGTGCAATAGTATTGTCATAGC | NA | NA | NA | NA | NA | NA | -13.4 | 0.0223 |
| mir-181a-3p | ACCATCGACCGTTGATTGTACC | NA | NA | NA | NA | NA | NA | -12.3 | 0.0435 |
| mir-181a-3p* | ACCATCGACCGTTGAGTGTACC | NA | NA | NA | NA | NA | NA | -12.3 | 0.0435 |
| mir-130-5p | CAGTGCAATATTAAAAGGGCAT | NA | NA | NA | NA | NA | NA | -8.7 | 0.0435 |
| mir-9-5p | ACTTTCGGTTATCTAGCTTTAT | NA | NA | -88.4 | NA | NA | NA | NA | NA |
| mir-137-5p | TTATTGCTTAAGAATACGCGTAG | NA | NA | -87.8 | 80.4 | 0.0017 | NA | NA | NA |
| mir-106a-5p | CAAAGTGCTTACAGTGCAGGTA | NA | NA | -58.8 | 12.3 | 0.0184 | NA | NA | NA |
| mir-106a-5p* | TAAAGTGCTTACAGTGCAGGTA | NA | NA | -58.8 | 12.3 | 0.0184 | NA | NA | NA |
| mir-3618-3p | TACATTAATGAAAAGAACAATGT | NA | NA | -56.9 | 49.2 | 0.0285 | NA | NA | NA |
| mir-217-5p | TACTGCATCAGGAACTGATTGGA | NA | NA | -41.4 | 29.6 | 0.0004 | NA | NA | NA |
| mir-92-5p | TATTGCACTTGTCCCGGCCTGT | NA | NA | -26.4 | 26.2 | 0.0012 | NA | NA | NA |
| mir-7-5p | TGGAAGACTAGTGATTTTGTTGT | NA | NA | -25.7 | 20.9 | 0.0110 | NA | NA | NA |
| mir-122-5p | TGGAGTGTGACAATGGTGTTTG | NA | NA | -20.1 | 10.5 | 0.0445 | NA | NA | NA |
| mir-23b-5p | GTGGTATCCCTGGCAATGTGAT | NA | NA | -19.2 | NA | NA | NA | NA | NA |
| mir-27b-5p | TTCACAGTGGCTAAGTTCAGTG | NA | NA | -16.7 | 13.8 | 0.0014 | NA | NA | NA |
| mir-221-5p | ACCTAGCATACAATGTAGATTTC | NA | NA | -16.3 | 7.3 | 0.0217 | NA | NA | NA |
| mir-96-5p | TTTGGCACTAGCACATTTTTGCT | NA | NA | -15.3 | 7.3 | 0.0043 | NA | NA | NA |
| mir-27e-5p | AGAGCTTAGCTAATTGGTGAGC | NA | NA | -14.2 | NA | NA | NA | NA | NA |
| mir-21-3p | CGACAACAGTCTGTAGGCTGTC | NA | NA | -13.1 | NA | NA | NA | NA | NA |
| mir-222-3p | AGCTACATCTGGCTACTGGGTCT | NA | NA | -11.3 | 12.3 | 0.0018 | NA | NA | NA |
| mir-200b-3p | TAATACTGCCTGGTAATGATGA | NA | NA | -11.1 | 5.1 | 0.0387 | NA | NA | NA |
| mir-146a-5p | TGAGAACTGAATTCCATAGATGG | NA | NA | -10.7 | 3.1 | 0.0354 | NA | NA | NA |
| mir-22a-5p | AGTTCTTCACTGGCAAGCTTTA | NA | NA | -9.9 | 8.2 | 0.0003 | NA | NA | NA |
| mir-199-3p | ACAGTAGTCTGCACATTGGTTA | NA | NA | -9.2 | 16.3 | 0.0003 | NA | NA | NA |
| mir-125a-3p | ACAGGTGAGGTCCTTGGGAACA | NA | NA | -9.2 | 15.6 | 0.0008 | NA | NA | NA |
| mir-200a-3p | TAACACTGTCTGGTAACGATGTT | NA | NA | -7.7 | 4.1 | 0.0106 | NA | NA | NA |
| mir-18a-5p | TAAGGTGCATCTAGTGCAGATAG | NA | NA | -7.6 | 5.2 | 0.0440 | NA | NA | NA |
| mir-93-5p | AAAAGTGCTGTTTGTGCAGGTAG | NA | NA | -6.8 | 6.6 | 0.0009 | NA | NA | NA |
| mir-23a-5p | ATCACATTGCCAGGGATTTCCA | NA | NA | -6.3 | NA | NA | NA | NA | NA |
| mir-203-3p | GTGAAATGTTTAGGACCACTTG | NA | NA | -6.3 | 3.9 | 0.0228 | NA | NA | NA |
| mir-30d-5p | TGTAAACATCCCCGACTGGAAGC | NA | NA | -5.9 | 7.0 | 0.0005 | NA | NA | NA |
| mir-142-3p | GTAGTGTTTCCTACTTTATGGA | NA | NA | -5.2 | NA | NA | NA | NA | NA |
| mir-184-3p | TGGACGGAGAACTGATAAGGGC | NA | NA | 3.8 | -3.1 | 0.0313 | NA | NA | NA |
| mir-183-5p | TATGGCACTGGTAGAATTCACTG | NA | NA | 4.2 | -13.9 | 0.0025 | NA | NA | NA |
| mir-196b-5p | TAGGTAGTTTCAAGTTGTTGGG | NA | NA | 4.3 | -4.3 | 0.0035 | NA | NA | NA |
| let-7c-5p | TGAGGTAGTAGGTTGTATGGTT | NA | NA | 5.2 | -3.9 | 0.0340 | NA | NA | NA |
| mir-133a-3p | TTGGTCCCCTTCAACCAGCTGT | NA | NA | 5.3 | -3.6 | 0.0387 | NA | NA | NA |
| mir-128-5p | CGGGGCCGGGGCGCTGTCTGAGA | NA | NA | 6.0 | -5.7 | 0.0003 | NA | NA | NA |
| let-7d-5p | TGAGGTAGTTGGTTGTATTGTT | NA | NA | 6.4 | -5.9 | 0.0008 | NA | NA | NA |
| mir-132-3p | TAACAGTCTACAGTCATGGCTAC | NA | NA | 16.5 | NA | NA | NA | NA | NA |
| mir-24-5p | TGGCTCAGTTCAGCAGGAACAG | NA | NA | NA | 51.4 | 0.0283 | NA | NA | NA |
| mir-29c-5p | TAGCACCATTTGAAATCGGTTA | NA | NA | NA | 43.1 | 0.0243 | NA | NA | NA |
| mir-192-3p | CCTGTCAGTTATGTAGGCCACTG | NA | NA | NA | 42.6 | 0.0479 | NA | NA | NA |
| mir-462-5p | TAACGGAACCCATAATGCAGCTG | NA | NA | NA | 16.4 | 0.0387 | NA | NA | NA |
| mir-103-3p | AGCAGCATTGTACAGGGCTATGA | NA | NA | NA | 7.3 | 0.0137 | NA | NA | NA |
| mir-126b-5p | ATTATTACTCACGGTACGAGTT | NA | NA | NA | 7.0 | 0.0006 | NA | NA | NA |
| mir-429-5p | TAATACTGTCTGGTAATGCCGT | NA | NA | NA | 6.7 | 0.0176 | NA | NA | NA |
| mir-214-3p | TACAGCAGGCACAGACAGGCAG | NA | NA | NA | 6.3 | 0.0228 | NA | NA | NA |
| mir-455-3p | TGCAGTCCATGGGCATATACAC | NA | NA | NA | 5.9 | 0.0096 | NA | NA | NA |
| mir-126-5p | CATTATTACTTTTGGTACGCGC | NA | NA | NA | 5.7 | 0.0041 | NA | NA | NA |
| mir-20-3p | ACTGCAGTGTGAGCACTTGAAGT | NA | NA | NA | 5.2 | 0.0178 | NA | NA | NA |
| mir-24-3p | GTTCCTGCTGAACTGAGCCAG | NA | NA | NA | 5.1 | 0.0031 | NA | NA | NA |
| mir-101-3p | TACAGTACTGTGATAACTGAAG | NA | NA | NA | 4.8 | 0.0228 | NA | NA | NA |
| mir-455-5p | TATGTGCCCTTGGACTACATCG | NA | NA | NA | 4.7 | 0.0032 | NA | NA | NA |
| mir-196-3p | CTACAACACGAAACTGTCTGA | NA | NA | NA | 4.5 | 0.0344 | NA | NA | NA |
| mir-489-5p | TGGTCGTATGTATGACGTCATT | NA | NA | NA | 4.2 | 0.0387 | NA | NA | NA |
| mir-145-5p | GTCCAGTTTTCCCAGGAATCCCT | NA | NA | NA | 4.0 | 0.0229 | NA | NA | NA |
| mir-30b-5p | TGTAAACATCCTACACTCAGCT | NA | NA | NA | 3.7 | 0.0242 | NA | NA | NA |
| mir-365-5p | TAATGCCCCTAAAAATCCTTAT | NA | NA | NA | 3.4 | 0.0216 | NA | NA | NA |
| mir-7132b-5p | GACTTGGTCAAAGCTCCTCAGC | NA | NA | NA | 3.0 | 0.0478 | NA | NA | NA |
| mir-26a-5p | TTCAAGTAATCCAGGATAGGCT | NA | NA | NA | -3.1 | 0.0452 | NA | NA | NA |
| mir-25-5p | CATTGCACTTGTCTCGGTCTGA | NA | NA | NA | -3.6 | 0.0166 | NA | NA | NA |
| mir-196-5p | TAGGTAGTTTCATGTTGTTGGG | NA | NA | NA | -4.1 | 0.0199 | NA | NA | NA |
| let-7a-5p | TGAGGTAGTAGGTTGTATAGTT | NA | NA | NA | -4.3 | 0.0183 | NA | NA | NA |
| mir-129-3p | AAGCCCTTACCCCAAAAAGCAT | NA | NA | NA | -5.4 | 0.0118 | NA | NA | NA |
| mir-1a-5p | ACATACTTCTTTATGTACCCAT | NA | NA | NA | -6.5 | 0.0137 | NA | NA | NA |
| new-miR-66 | ACTGTCACCCTGATATATTACT | NA | NA | 38.4 | NA | NA | NA | NA | NA |
| new-miR-34 | ATACTGTACATTTGTGATTGAG | NA | NA | 6.9 | NA | NA | NA | NA | NA |

**Supplementary Dataset 1D**: Real time PCR validation of fold change of 12 microRNAs DE between high and low muscle yield group. RNA-Seq and qPCR show consistency in expression pattern of microRNAs. For qPCR analysis, total RNA from 16 individual fish from each high ranked and low ranked family (n=32) was used. Error bars on qPCR data represent standard deviation. Fold change was statistically significant by RNA seq (FDR-p>0.05) as well as by qPCR (p>0.05). For qPCR data p value was calculated using non-parametric Mann-Whitney U test.


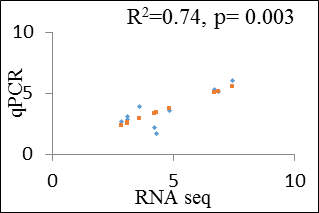

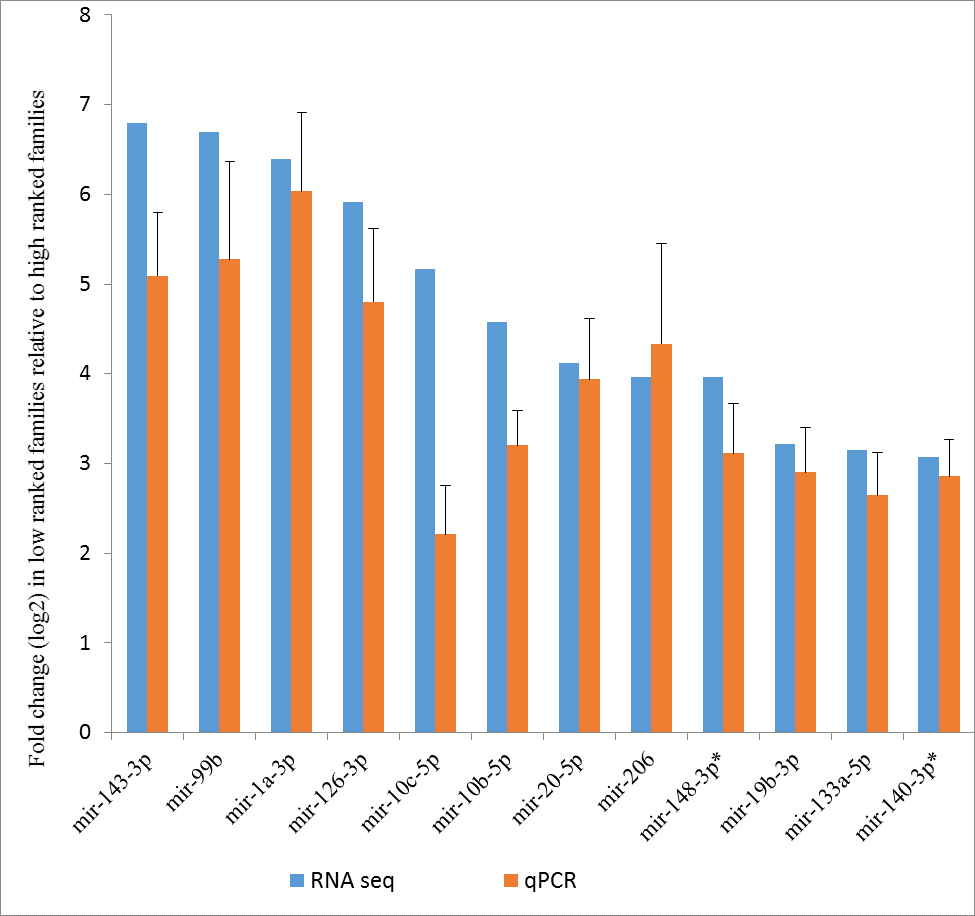


**Supplementary Dataset 1E**: Multidimensional scaling plot on N x N matrix of genome-wide IBS pairwise distances showing fairly homogeneous sample with no obvious population stratification or significan clustering.

**
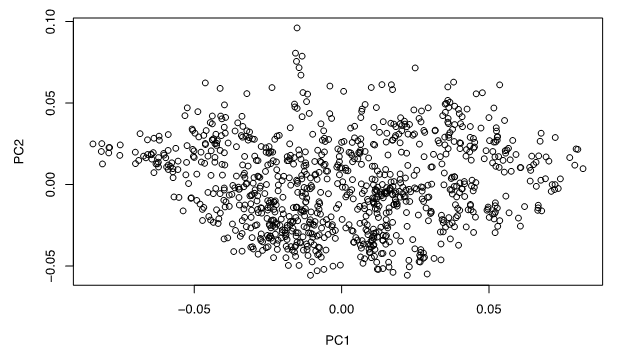
**
